# PReS-FINAL-2182: The role of murine rage in innate immune responses and inflammation: a systematic approach

**DOI:** 10.1186/1546-0096-11-S2-O17

**Published:** 2013-12-05

**Authors:** T Wirth, C Kessel, T Weinhage, P Becker, N Nippe, D Foell

**Affiliations:** 1 Department of Pediatric Rheumatology and Immunology, University Children's Hospital Muenster, Muenster, Germany; 2 Department of Dermatology, University of Muenster, Muenster, Germany

## Introduction

The receptor for advanced glycation end products (RAGE) is a multi-ligand receptor expressed on various cells which interacts with a diverse class of ligands, e.g. 'danger signals' such as neutrophil-derived S100A12. RAGE has been implicated in the pathogenicity of various inflammatory diseases. However, the exact role of RAGE has not been sufficiently defined. Our recent data on S100A12-induced activation of monocytes points to a modulatory rather than pro-inflammatory function of human RAGE.

## Objectives

To assess the role of RAGE in a more systematic way, we generated RAGE-/- mice and analyzed immune cell functions *in vitro*, followed by murine models of *staphylococcus aureus *infection (dermal immunity), chemically induced colitis (mucosal immunity), lethal inflammatory liver injury (septic shock) and collagen induced arthritis (autoimmunity) comparing RAGE-/- and C57BL/6 wildtype (wt) mice.

## Methods

Reactive oxygen species (ROS) production, phagocytosis and cytokine production of bone marrow derived monocytes were measured using flow cytometry. *Staphylococcus aureus *(*S. aureus) *infection was induced by footpad injection. Acute and chronic colitis were induced chemically by dextran sodium sulfate (DSS) solution. For the inflammatory liver injury model, mice were challenged with D-galactosamine (D-Gal) along with lipopolysaccharide (LPS). Arthritis was induced by injection of heterologous type II collagen (CII).

## Results

We observed no differences of cellular immune function (ROS and cytokine production, phagocytosis) in RAGE-/- compared to wt monocytes. Male, but not female RAGE-/- mice showed more footpad swelling and bacterial dissemination in the *S. aureus *infection model. In the DSS colitis model we observed no significant differences between the two strains, whereas RAGE-/- mice were significantly protected from lethal D-Gal/LPS induced liver injury. RAGE-/- and wt mice develop arthritis at similar clinical scores and incidence with no detectable differences in type II collagen autoantibody levels. However, in female animals there is a strong tendency towards aggravated disease, less remission and higher disease penetrance.

## Conclusion

Overall, the contribution of RAGE seems to largely depend on the disease model and cell type studied. While no overall differences with respect to immune cell activities were observed, a striking gender specific effect of RAGE seems to be involved in some conditions.

## Disclosure of interest

None declared.

